# Towards construction of embankment on soft soil: a comparative study of lightweight materials and deep replacement techniques

**DOI:** 10.1038/s41598-024-77587-0

**Published:** 2024-11-28

**Authors:** Fathi M. Abdrabbo, Khaled E. Gaaver, Amr Z. Elwakil, Shahinaz A. Khalifa

**Affiliations:** 1 Structural Engineering Department, Faculty of Engineering, Alexandria University, Alexandria, Egypt; 2 Faculty of Engineering, Alexandria University, Alexandria, Egypt

**Keywords:** Embankment, Lightweight, Soft clay, Sand pile, Stone column, Deep mixing, Plaxis, Engineering, Civil engineering

## Abstract

Planning embankments demands comprehensive studies to select suitable materials, enhance soil stability, ensure optimal performance, and comply with building code requirements and sustainability standards. This study offers an evaluation of various alternatives and their effectiveness for constructing embankments on weak soil using the 2D finite element software Plaxis 8. It highlights the convergence of different techniques, offering flexibility in selecting the optimal strategy for projects. The behaviour of multilayer clayey soil under an embankment of lightweight filling materials such as mixed sawdust or geo-foam and that carrying an embankment of traditional fill material improved by deep replacement techniques like concrete piles (CP), deep-mixing columns (DMC), stone columns (SC), and sand piles (SP) were compared considering factors like stress distribution, pore water pressure, and settlement of the soil. The results demonstrate that lightweight materials reduced settlement by 11–98% and stress by 5–89%, while deep replacement techniques reduced settlement by 8.5–75% and stress by 44–88%. Notably, the study underscores the effectiveness of DMC in promoting soil reuse compared to CP.

## Introduction

Clay soils pose serious challenges to construction projects globally, causing significant economic losses that vary by region. Understanding soft soil influenced by factors such as the mineralogical features of clay minerals (like illite, kaolinite, and montmorillonite), high surface areas, water absorption, and expansion, which can lead to foundation instability and ground movement, is crucial for mitigating such issues. So, one of the main objectives for geotechnical engineers is to improve the characteristics of highly compressible soft soil to withstand the stress exerted by structures. Improving the soil beneath embankments involves various techniques aimed at densifying and enhancing soil characteristics for better productivity and sustainability. Key approaches include chemical amendments, mechanical interventions like vibrio-compaction, and water management. Furthermore, several scholars have recommended considerations or approaches for constructing embankments on soft soils to help determine the best strategies for enhancing the carrying soil. Others have predicted techniques for simulating embankment construction in geotechnical centrifuges using various assumptions to improve measurement accuracy^1^.

Employing lightweight materials such as sawdust and geo-foam in embankment construction can reduce the weight and stress on the soil, allowing it to carry the embankment without requiring additional soil improvement. These materials are easy to install without specialized tools^2,3^ and help in reducing excess pore water pressure, leading to long-term soil consolidation^4^ that reduces the settlement and stress of the surrounding soil. Also, one of the oldest methods for improving soft soil under the embankment is replacing the top of soft soils with granular soils with or without reinforcement layers. Which enhances the bearing capacity and reduces the settlement^5^ Replacing soil under a shallow foundation supporting light structures can significantly reduce construction costs, although the replacement depth is usually determined through experience^6^. Furthermore, replacing the weak soil with biopolymer-modified soil by adding stabilisers or a geogrid layer decreases stress and displacement in both lateral and vertical directions^7,8^ without any adverse environmental effects^9^.

However, for embankments filled with traditional or heavy material, applying deep replacement techniques such as CP, SC, SP, and DMC can help enhance soil behaviour more than replacing the top layers. Multiple studies have focused on understanding the behaviour and effectiveness of various deep replacement techniques. However, these studies often focus on determining vertical loads and stress at embankments supported by CPs based on complex calculations that cannot predict settlement or excess pore water pressure. Attempts are made to simplify and generalize these calculations in codes or specifications^10,11^, which analyze embankments supported by CPs. Moreover, by comparing different soil improvement methods beneath an embankment, it can be noted that the potential slip surfaces for embankments built on soft soil are circular, with compression failure under the crest, bending failure under the shoulders, and tensile or bending failures near the toe^12^. And, each CP often fails by bending but has a different failure strategy. So, the failure modes of piled embankments can’t be predicted from site tests^12,13^. However, several analytical models expected the load transfer mechanism from the embankment to the supported piles and carried soil based on soil arching effect via laboratory tests assuming that piles were arranged in a square^14^ or triangular^15^ pattern.

Furthermore, evaluating SCs supporting embankments and developing an analytical approach to calculate stress and loads transferred to the surrounding soil was investigated^16^. Where the shearing characteristics of SCs or SPs have a crucial impact on soil improvement and enhance the soil’s load-carrying capability. SCs or SPs boost bearing capacity, minimise post-construction settlement and lateral movement, and improve slope stability and liquefaction management^17,18^, they accelerate clay consolidation and pore water pressure dissipation^19^. Furthermore, the combination of reinforcing layers with SC considerably boosts the soil’s bearing capacity by up to 8.0 times^20^. Additionally, combining SC and DMC enhances safety, reduces consolidation settlement, and provides drainage performance similar to SCs^21^and derived a method to estimate the degree of consolidation in soft soil improved by DMC and supporting a rigid footing^22^. The effectiveness of treating soft clay with DMC techniques primarily depends on the lime type and chemical reactions between the lime and soil minerals^23^. By comparing the performance of DMC and SC-supported embankments, the results show that the addition of DMC below the embankment reduces settlement and lateral displacement by 73% and 38%, respectively, compared to SCs^24^.

The current study compares the effectiveness of various methods for improving soft soil for earth embankment construction. It uses 2D finite element software Plaxis 8 to simulate the behaviour of soft soil beneath an embankment constructed using lightweight filling materials such as soil mixed with sawdust or geo-foam and the soil improved by deep replacement techniques like Cp, DMC, SC, and SP carrying heavy embankment. The investigation includes analyzing settlement, stress distribution, axial tensile force acting on the geosynthetic layer, and excess pore water pressure.

### Case study and soil formation

**Fig. 1:**
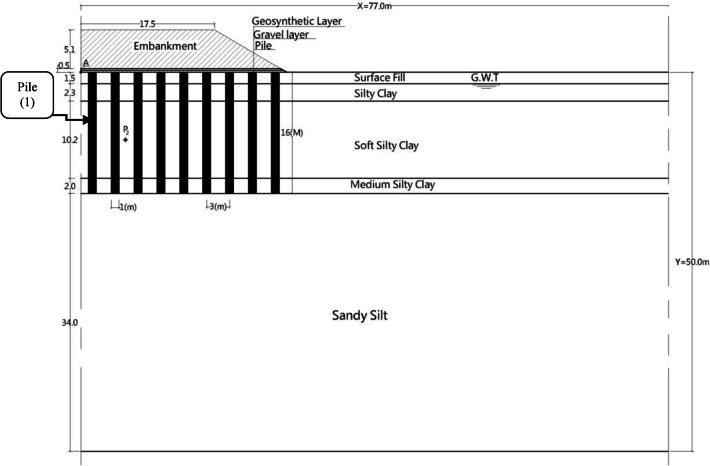
Case study^25^.

The site formation considered in this numerical analysis is located in a northern suburb of Shanghai, China^25–27^, as shown in Fig. 1. The figure illustrates an embankment of 5.6 m total height, divided into 5.1 m of initial filling material (IFM) of density 18.5 kN/m^3^ density and 0.5 m gravel cushion reinforced with a geosynthetics grid, was constructed in two layers over 55 days. This embankment was built on multi-layered soil consisting of 1.5 m of surface fill, 2.3 m of silty clay,10.2 m of soft silty clay,2 m of medium silty clay, and an extended layer of sandy silt. The groundwater table (G.W.T) was 1.5 m below the ground surface, and the constitutive parameters of the soil are presented in Table 1. The soil was supported by CPs arranged on square patterns of 16 m length, 1 m diameter and 3m spacing in both directions. The changes in the underlying soil properties that would occur due to the construction of piles or reinforced gravel cushions were not considered.

**Table 1:** The model parameters^25^.

Element name	E (MPa)	γ (kN/m^3^)	Φ	C (kPa)	ʋ	K (m/day)	Cam clay parameter			J (kN/m)
							λ	k	m	
Surface fill	7	20	28	15	0.3	–	–	–	–	–
Silty clay	–	20	–	10	0.35	8.64 × 10^−4^	0.06	0.012	1.20	–
Soft silty clay	–	17	–	20	0.40	4.32 × 10^−4^	0.15	0.03	0.95	–
Medium silty clay	–	20.5	–	35	0.35	4.32 × 10^−4^	0.05	0.01	1.10	–
Sandy silt	–	20	20	4	0.35	4.32 × 10^−4^	0.03	0.005	0.28	–
Embankment fill(IFM)	20	18.5	30	10	0.30	–	–	–	–	–
Gravel cushion	20	18.5	40	10	0.30	–	–	–	–	–
Concrete Pile	20,000	24	–	–	0.2	–	–	–	–	–
Geosynthetic	–	–	–	–	0.3	–	–	–	–	1180

E: Young’s modulus (kN/m^2^), γ: Unit weight of the soil (kN/m^3^), J: linear Elastic modulus (kN/m), Φ: Friction angle (^0^), c: Cohesion (kN/m^2^), υ: Poisson’s ratio, K: Permeability, λ, k and m: Cam Clay model parameters.

### Numerical model assumptions

**Fig. 2:**
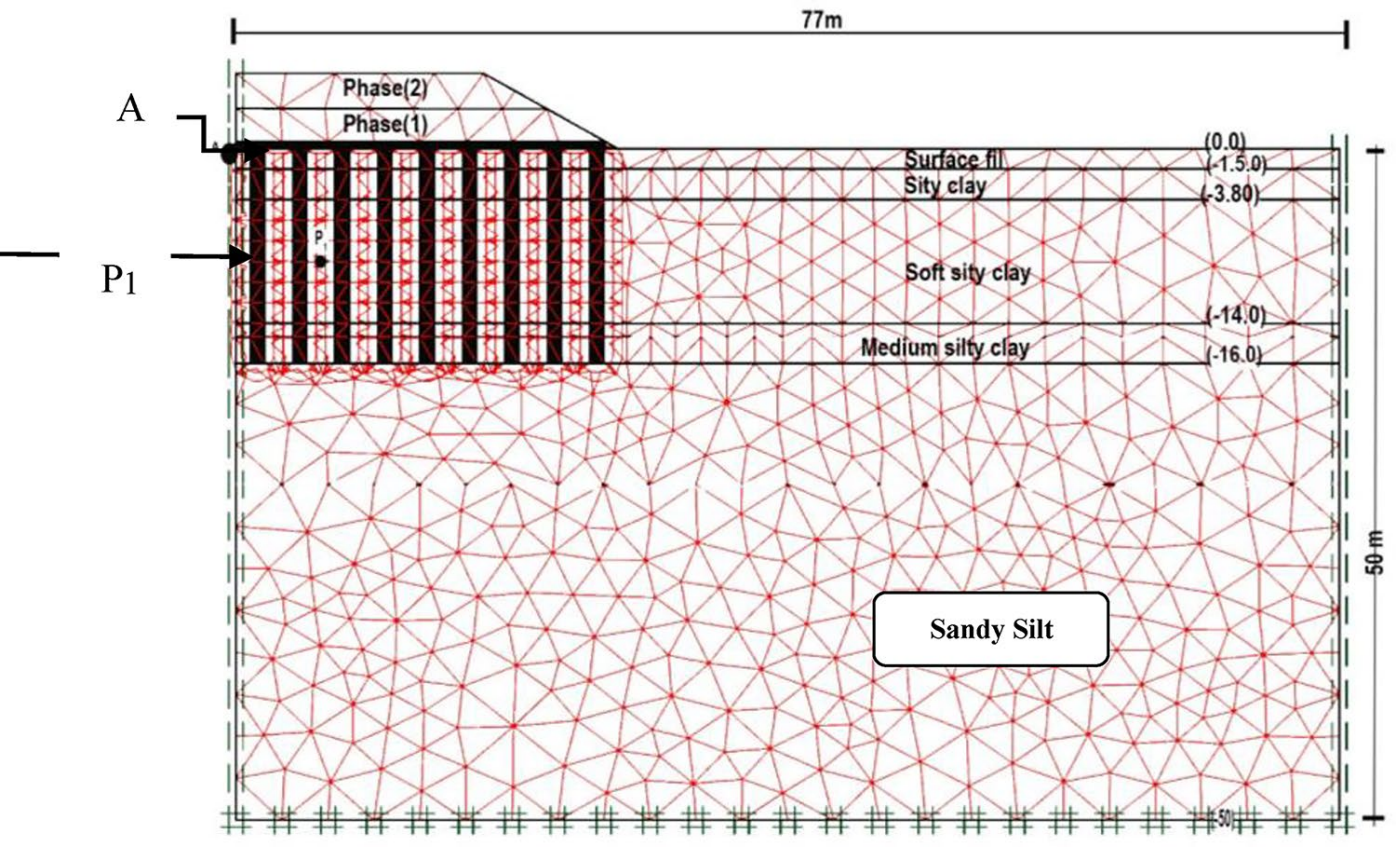
Numerical model.

**Fig. 3:**
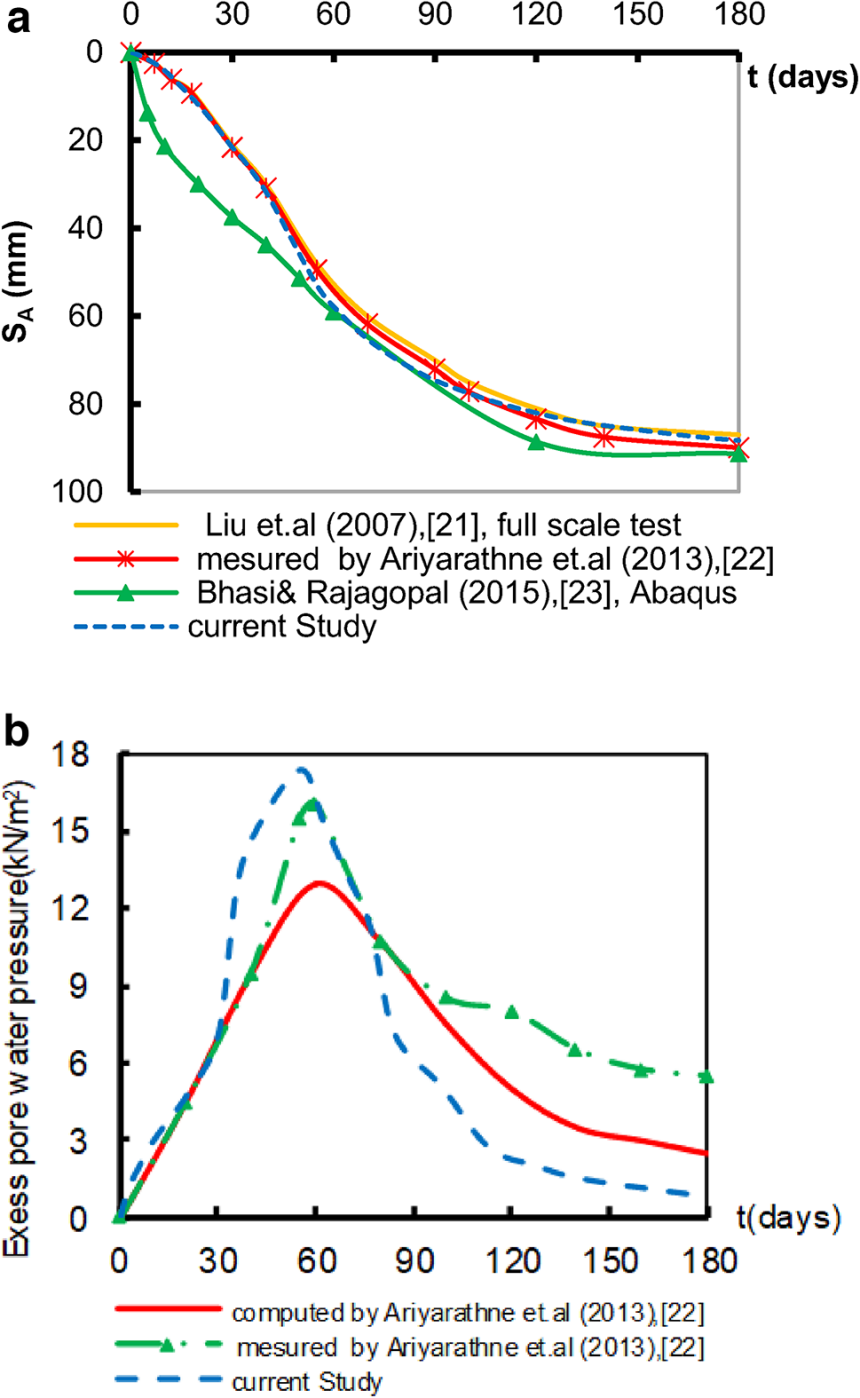
Model results for validation. (**a**) Settlement at point A versus time t. (**b**) Excess pore water pressure at point P_1_versus time t.

Plaxis program version 8 utilizing 2D plane strain assumptions were employed to simulate the numerical model, Fig. 2. The program simulates complex soil behaviour and provides numerous constitutive models appropriate for geotechnical applications, including soft to stiff soils, staged loading, and soil interaction with structural components^28^. The model was conducted using sensitive studies, including dimensions and time domain, constraints, model type, element, and mesh size, which were then verified with measured and computed data introduced in previous works, as shown in Fig. 3.

Soil and embankment clusters were simulated as 15-node triangular elements. A standard fixity condition was applied that provides a simple and effective boundary condition for modelling soil-structure interactions and is perfectly fixed at the boundary, allowing for accurate simulation. The study focused on half of the soil and embankment domain, with the side boundary positioned at a distance of 77 m from the centerline of the embankment and extending to a depth of 50 m below the surface. The embankment filling materials and the gravel cushion layer were simulated using the Mohr coulomb-undrained model. Mohr coulomb model is widely applicable to various soil types and conditions, making it a versatile choice for modelling different geotechnical scenarios due to its simplicity, efficiency, and adequate representation. Furthermore, many engineering codes were based on the assumption of the Mohr-Coulomb model^28^. While the soil layers were simulated as Soft Soil-drained models. The model predicts settlement and deformation in clayey soils by effectively representing their compressibility, volume changes, and time-dependent behaviours such as consolidation, which impact the long-term stability and settlement of structures. It also facilitates layered soil analysis, providing a more precise depiction of clay layers and their interactions with structures. With its advanced parameter considerations, the Soft Soil model ensures reliable analysis that complies with safety and design standards^28^. The geosynthetic reinforcement layer was represented by adding a geogrid element in Plaxis. Which is defined as a thin construction with axial stiffness, capable of withstanding tensile forces only when axial strain occurs. It is considered a linear-elastic material that adheres closely to the surrounding soil^28^.

The deep replacing material (piles) were represented as embedded piles raw, suggested by Plaxis 8 which models pile-soil interaction using beam elements for piles^29^, as a non-porous linear elastic model that provides a reasonable approximation for many engineering applications, and it is essential to recognize the limitations of this simplification, especially in environmental geotechnics^28^. To establish the connection between the piles and the soil nodes, an out-of-plane interface called the “Rigid Interface, R = 1” was used in the studied model. For accurate calculations and computational efficiency, a very fine mesh was employed and the groundwater table was assigned.

The calculation is divided into three phases. The installation of embankment layers was represented in two phases, Phase 1 represented the construction of the gravel cushion with a geosynthetic reinforcement layer and 2.6 m of the embankment height over 30 days, while Phase 2 simulated the placement of an additional 2.5 m of the embankment height over 25 days. Phase 3 looked at how consolidation affected the embankment’s behaviour for 745 days, examining the embankment’s behaviour over two years starting at the construction beginning.

Figure 3a shows that the obtained results of settlements are consistent with the field test^25,26^. At the same time, the results underestimate the settlement by about 5% when compared to the results of the numerical analysis^27^. Additionally, the excess pore water pressure versus time is determined at point (P_1_). Figure 3b is a comparison of the obtained results with the measured values^25,26^. The achieved results reasonably match the computed values, but they are less than the measured value before finishing the embankment installation. Nevertheless, at the end of the installation process over (55) days, the excess pore water pressure achieved the maximum value with a variation between the results. This might be attributed to the installation of piles, which reduces the phase of non-Darcian flow^30^. At the end of construction time, the excess pore water pressure started to dissipate. Additionally, the findings of the present study revealed a 1.0% underestimation of stress compared to the field data and a 30.2% underestimation compared to the numerical analysis^27^. Furthermore, the CP load obtained from Plaxis was 2.8% higher than the values reported by the measured data^25^ and 4.0% lower than the computed results^27^. Finally, the axial tensile force acting on the geosynthetic layer was confirmed using the calculated values in previous studies^25,27^ and it can be concluded that the Plaxis calculation demonstrates convergence with these values in Table 2.

**Table 2:** Stress and the load validation.

Reference	Stress on soil (kPa)	Load on pile 1 (kN)	Axial tensile force (kN/m)
Liu et al.^25^	37.2	458.3	13
Bhasi and Rajagopal^27^	52.8	490.4	13.1
Current study	36.85	471.1	13.67

This study utilizes a 2D finite element program Plaxis 8 to present a comprehensive analysis of the effectiveness of several techniques for constructing earth embankments on soft soil, focusing on two strategies. The first is based on reducing the weight of the embankment using lightweight materials, such as soil mixed with sawdust or geo-foam for constructing the embankment. Table 3 summarizes the characteristic properties of the investigated materials. The second, focused on improving the characteristics of the soft soil layer through shallow and deep replacement techniques. Deep replacement of soil includes stone columns (SC), sand piles (SP), and deep-mixing columns (DMC), see Table 3. SCs and SPs, which are vibro-replacement techniques of vertical columns of compacted aggregate that are formed within the soils to be improved. DMC is created by mixing cement or lime with soil to produce a stronger column capable of supporting loads and reducing anticipated settlements. Deep mixed columns exhibit excellent cohesiveness, thereby reducing groundwater flow and minimizing embankment settling (S). The various replacement cases have the same dimensions and arrangement of CP.

**Table 3:** Characteristic properties of the investigated material.

Element	E (MPa)	γ (kN/m^3^)	Φ	C (kPa)	ʋ	K (m/day)	References
Replacement soil	30	20	35	20	0.3	0.007	Das^31^
Stone column, SC	100	18.50	45	10	0.20	0.07	Das^31^
Sand pile, SP	25	20	35	25	0.30	0.007	Das^31^
Deep mixed soil, DMC	1250	16.31	32	340	0.20	--	ASTM^32^
Sawdust	0.85	4	32	30	0.05	0.0864	Ogunribido^33^
Geofoam	3.76	1	32	110	0.09	0.001	ASTM ^34^

### Improvement techniques assumptions and results

**Fig. 4:**
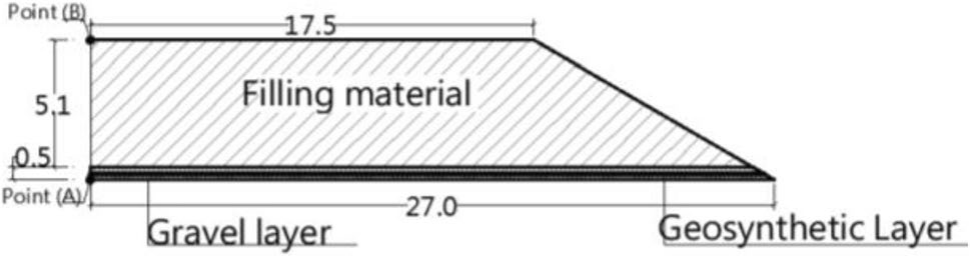
Detailing of the embankment height^25^.

Numerous numerical models were conducted to evaluate the assumed studied model, Fig. 1, on native soil. An embankment consisting of 0.5 m of gravel cushion of unit weight of 18.5 kN/m^3^and 5.1 m of initial filling material (IFM) of unit weight 18.5 kN/m^3^, Fig. 4, on this native soil, was analyzed through the numerical program. The analysis found that the soil cannot withstand this height without a need to improve its properties. So, several models were conducted to determine the IFM maximum height or critical height (H_Cr_) which can be constructed on native soil without requiring improvement and inducing a settlement that does not exceed the allowable value which less than 600 mm^35^. The assumed IFM height and corresponding settlement are summarized in Table 4. The analysis revealed that the height of IFM should not exceed 3.0 m, which represents 60% of the supposed IFM height. Therefore, building 5.1 m of embankment height necessitates using low-density construction materials or needs soil improvement.

**Table 4:** The studied height and corresponding settlement after 800 days.

IFM height (m)	S_A_ (mm)
2.60	428.10
2.70	446.12
2.80	463.27
2.90	482.12
3.0	516.58
3.10	621.12

### Lightweight fill

Studying the using lightweight materials for filling embankments is crucial for improving stability, reducing costs, enhancing environmental sustainability, and addressing the unique challenges posed by soft soils like collapse or non-allowed settlement. It is essential to try reducing the load on the soil before considering alternative improvement methods. This option may be constrained by the compressibility that can occur in the embankment body under the applied loads, depending on its intended use. Therefore, it is essential to standardise the compressibility calculations of the lightweight embankment and establish clear standards for the use of these materials in building codes and specifications.

Lightweight materials, such as sawdust and geo-foam, can be used to partially or completely replace IFM materials to reduce stress on soft soil. Sawdust, a loose material with a diameter between 10 and 30 μm^36^, derived from cutting and burning wood, has a density ranging from 370 to 415 kg/m^3^^33^. However, using sawdust may lead to compressibility issues in the embankment, and appropriate treatment to prevent fire hazards. On the other side, geo-foam made by producing expanded or extruded polystyrene (EPS/XPS) into large, lightweight blocks, has a density ranging from 1.92 to 2.88 kg/m^3^^34^, representing only 1–2% of the soil density^37^. Geo-foam reduces settlement in geo-structures and offers various applications such as drainage, thermal insulation, lightweight fill, compressible inclusions, and green roof fill. Similar to sawdust, geo-foam requires treatment to prevent fire hazards and should not be exposed to petroleum solvents, as it loses load-bearing capacity and may experience buoyancy-related uplift forces.

**Fig. 5:**
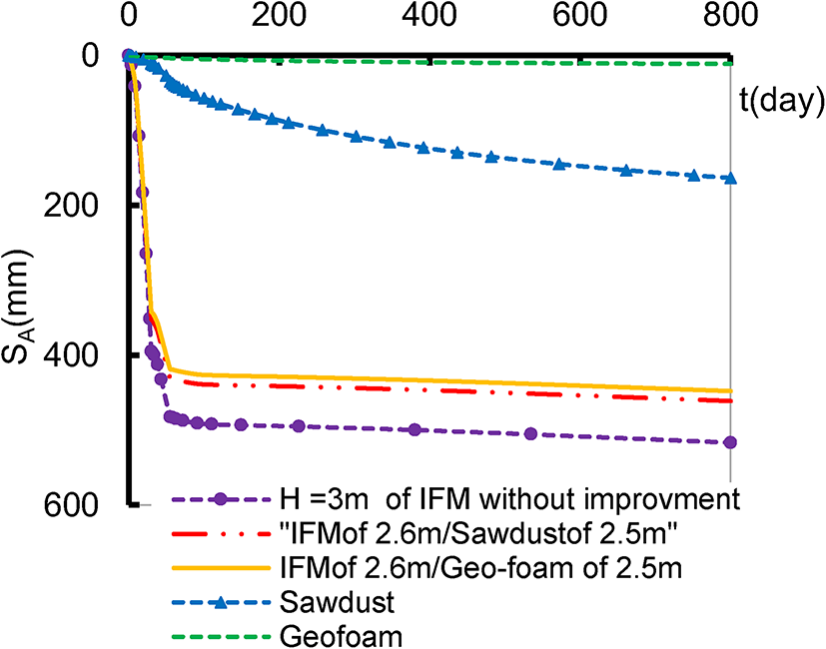
The effect of embankment fills on the settlement at point A.

**Fig. 6:**
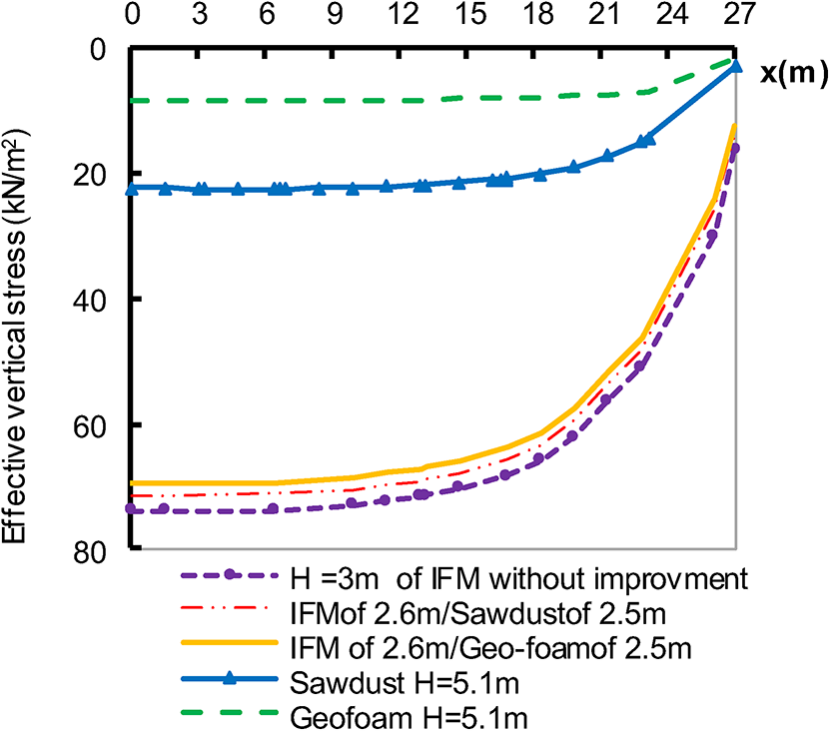
Effective vertical stress underneath the embankment.

To address the issue of embankment weight, four sets were studied involving the aforementioned lightweight materials and compared by the settlement and stress of the embankment constructed up to H_Cr_ on non-improved soil. In two cases, only half, or partially filling, of IFM was replaced with lighter materials. The filling weight was divided into 2.6 m of IFM and 2.5 m of either geo-foam or sawdust. Additionally, the other two cases were examined, assuming the possibility of filling the total embankment height with geo-foam or sawdust. The embankment filling materials IFM, geo-foam or sawdust) were simulated using the Mohr coulomb-undrained model. The settlement at point A of these four cases is illustrated in Fig. 5 as well as the effective vertical stress underneath the embankment at various positions (x) in Fig. 6. The settlement reduction ratio (SRR %) is introduced as shown in Eq. (1). Table 5 shows the obtained SRR % due to employing the different studied lightweight materials.

1 $$\text{SRR}\% = (\text{S}_{0}-\text{S}_{\text{A}})/\text{S}_{0} \times 100$$

where: SRR% is the settlement reduction ratio factor, So is the settlement at point A of 60% of the embankment height constructed on non-improved soil after 800 days, and S_A_ is the settlement of point A soil after soil improvement after 800 days.

The results demonstrate that, even though the building embankment with lightweight material of 5.1 m height, there is a reduction in the settlement and stress for all the studied cases compared to the settlement that occurred due to the construction of 3 m of the embankment height using IFM height. In the partial filling with lightweight material, the settlement was reduced by approximately 11%. The full filling with lightweight material exhibited a notable reduction in settlement by 98% for geo-foam and 68% for sawdust. In addition, the stress affected the soil along the embankment base is illustrated in Fig. 6. The finding illustrates a reduction by 25.26% in the stress in the partially filled with geo-foam and 18% in the case of partially filed sawdust. Also, for the full-filled cases, the stresses reduced by 88.55% for Geo-foam and 70% for sawdust. These highlight the importance of accurately specifying construction materials based on the purpose of the project and the soil’s bearing capacity to prevent the collapse of weak or soft soil.

Given these results, the use of lightweight fill could be a practical solution for saving time and simplifying the construction process. However, it is important to consider the compressibility of the embankment body, as it can affect the feasibility of using lightweight fill. These materials usually have lower stiffness compared to traditional filling materials and may have internal voids. This leads to compression under the acting loads as these voids rearrange under pressure, which increases the settlement of the top surface of the embankment. Compressibility (comp %) illustrated in Table 5 represents the ratio of the embankment body’s subsidence (S_S_), see Eq. (2), to its height H.

2 $$\text{Comp}\% = \text{Ss}/\text{H} \times 100$$

where: Ss = S_B_- S_A_. S_B_ is the settlement at the embankment surface (mm) after 800 days and S_A_ is the settlement at the embankment base (mm) after 800 days, and H is the fill height (m).

The findings in Table 5 indicate that the comp% for filling the embankment with lightweight material exceeds threefold compared to partially filling the height with the same material and IFM. Incorporating IFM effectively restricts surface deformation of the embankment, albeit resulting in settlement and heightened stress on the soil. Moreover, the research assesses the axial tensile force (kN/m) applied to the geosynthetic layer in Table 5, which aids in mitigating the arching effect within the soil. This layer effectively manages tension and stress and redistributes forces across the soil surface. Geosynthetics do not prevent soil settlement but rather distribute loads evenly over the surface. The axial tensile force (kN/m) is influenced by material types and increases with higher embankment weights.

### Shallow replacement of the soil

Replacing the top part of soft soil beneath the embankment with granular soil improves bearing capacity and reduces settlement. But, the thickness of the replacement layer needs to be carefully studied^7^. Therefore, a set of models was conducted to replace the top portion of the soft soil with a stronger one, Table 3. In each model, the thickness (thr) and width (B) of the replacement layer were increased by approximately 2 m. The results indicated that the minimum replacement thickness required to support the full height of the embankment is 8.0 m. However, implementing this method poses significant challenges, including time-consuming procedures, demanding precautions, and the need for a dewatering process during soil replacement and compaction. As a result, this method is considered impractical for the proposed case study.

### Deep replacement of the soil

**Fig. 7:**
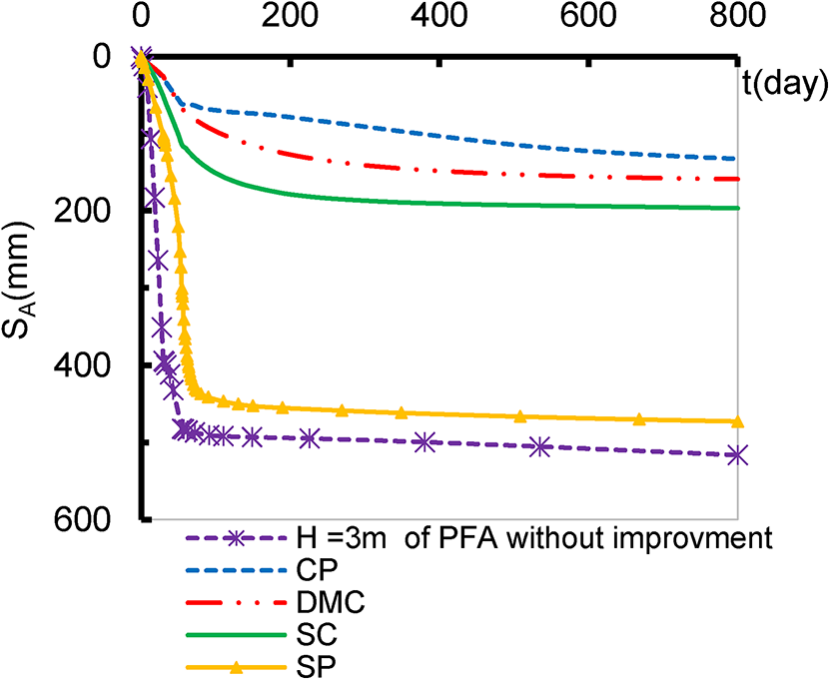
The effect of deep replacing material.

**Fig. 8:**
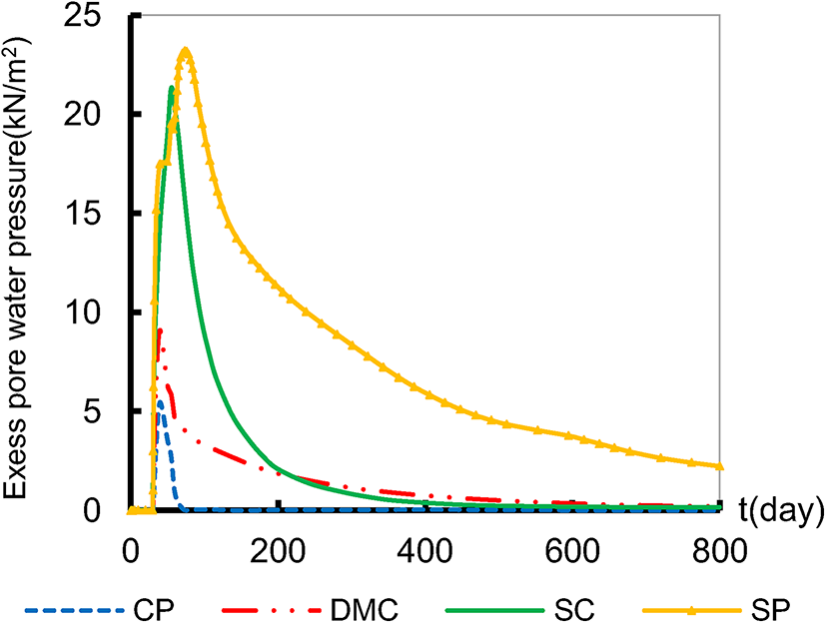
Excess pore water pressure at A.

**Fig. 9:**
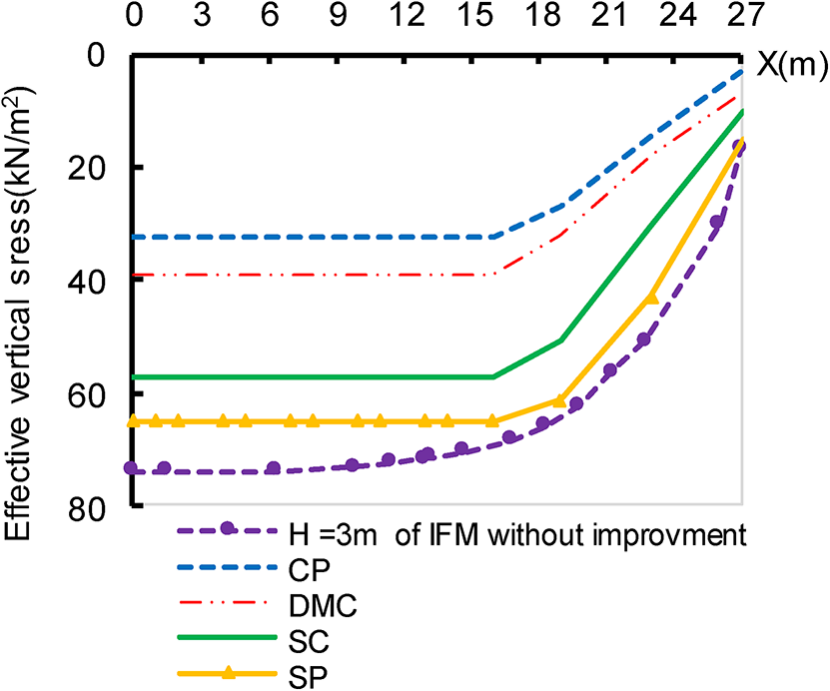
Effective vertical stress on the soil beneath the embankment, after 800 days.

**Fig. 10:**
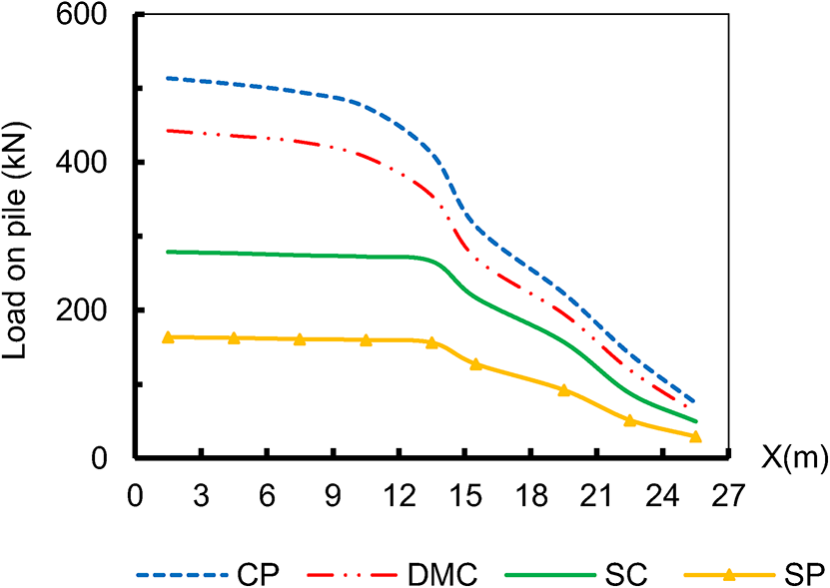
Vertical load on piles beneath the embankment, after 800 days.

Deep replacement techniques, which involve substituting soft soil with high-strength materials at significant depths, are crucial for improving load-bearing capacity, reducing settlement, enhancing soil properties, and ensuring the stability and performance of foundations in challenging soil conditions. The effectiveness of various deep replacement materials in enhancing soil stability and load-bearing capacity was evaluated to, identify the most suitable solutions for different applications, and examine the durability and long-term behaviour of these materials. Figures 7, 8, 9 and 10 present a comparison between the behaviour of the embankment supported by different deep replacement materials and that constructed up to H_Cr_ on non-improved soil. The validation analysis was conducted on concrete piles (CP), having a length of 16 m, 1 m of diameter and spaced at 3 m. Then, CP is replaced with different alternative deep-replacing material types like stone columns (SC), sand piles (SP) and deep mixed soil columns (DMC) having the same dimensions and geometry illustrated in Fig. 1. SPs, SCs, and DMCs were simulated by Mohr-Coulomb-drained models as embedded pile raw having interface values of 0.7, 0.8, and 0.9 respectively, according to Plaxis 8 user manual equations for calculating the pile interface^28,38^. Figure 7 illustrates that settlement decreases with the reduction of the material stiffness, and the cohesion. DMC has less settlement compared to SPs and SCs. The settlement of the embankment supported by DMC is almost equal to the obtained by using CP. Also, by comparing SRR%, Table 5, of each replacing material type and the settlement exhibited due to H_Cr_ of IFM, it was found that the settlement reduced by ratio varied between 8.5% and 74.29%.

Also, the excess pore pressure dissipation controls the settlement that occurs on the supported clay soil as well as the stress acting on it. Figure 8 shows that the excess pore water pressure develops over 55 days for CP and DMC and over 78 days for SC and SP before reaching its maximum value and starting to dissipate. The drop in excess pore water pressure has a significantly highest dissipation value in the SP and SC. This can be attributed to the increased permeability due to SC and SP. Also, the effect of deep-replacing material on the excess pore water pressure–time relation is reflected in the settlement of the embankment, Fig. 7. Despite the convergence of excess pore water pressure dissipation values in the SC and SP, which helps to accelerate the consolidation settlement of the surrounding soil, the increasing the shearing properties of SC helped in strengthening the soil and constrained unaccepted settlement.

Figure 9 shows the relationship between effective vertical stress underneath the embankment after 800 days and horizontal distance. The figure demonstrates that DMC has a convergent behaviour of CP. The stress on the soil supported by SC and SP is increased to 1.77 and 2.02 times, respectively, compared to the stress of the soil in the case of employing CP. It can be attributed to changes in the shearing parameter, which controls how stress is distributed and transferred via the deep-replacing material and the soil. The figure also indicates that the stress imposed directly on soil is near a constant value for each replacement type. The more rigid the material, the less stress is imposed directly on the soil. This behaviour may explain the results of excess pore water pressure illustrated in Fig. 8. Also, Comparing the findings extracted in Fig. 9 with the non-improved case, there is a reduction in soil stress to 43.56%, 52.12%, 76.52%and 87.88% for CP, DMC, SC, and SP respectively.

**Fig. 11:**
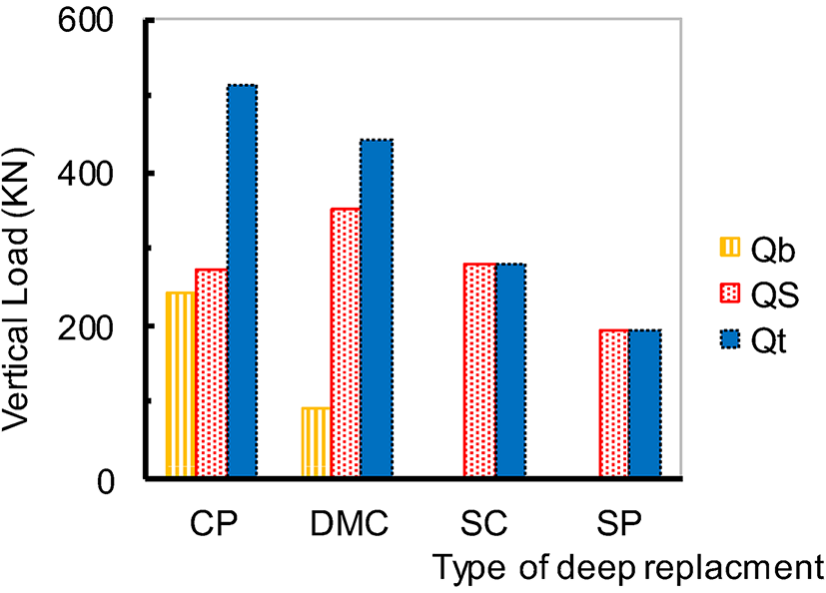
Loads transfer mechanism for Pile 1.

Moreover, Fig. 10 shows that, the load distribution on each pile beneath the embankment. The load distribution demonstrates that the piles carry most of the loads on the soil and assist in transferring stress through the soft soil layers. The vertical load per pile decreases as the distance from the embankment centreline increases. Also, it was found that the properties of the pile material control the load transfer mechanism through the soil layers. Figure 11 illustrates the pile’s load Q_t_ transfer mechanism at the embankment’s centre, pile 1. The figure reveals that CP and DMC transferred the load via the soil layer depending on both bearing Q_b_ and friction Q_f_ characteristic properties. SP and SC acted as friction piles. However, DMC increases the strength of the soil and provides bearing resistance^39^. In Table 5, the average axial tensile strength of the geosynthetic layer shows an increase as the modulus of elasticity of the deep replacement decreases. This phenomenon may be attributed to the higher generation of arching stresses within the soil, where fewer loads are transferred through various types of deep replacement as its modulus of elasticity decreases. Also, as time progressed over 180 days and 800 days, there was a noticeable decrease in stress applied to the soil and the geosynthetic layer, while the load carried by the structure increased. This observation underscores the time-dependent nature of analyzing structures constructed on soft soil.

**Table 5:** SRR%, Comp% and the axial tensile force acting on the geosynthetic layer after 800 days.

Improvement method	S_0_ (mm)	S_A_ (mm)	S_B_ (mm)	S_S_ (mm)	SRR%	Comp%	Axial tensile force (kN/m)
Geo-foam	516.58	11.18	69.01	57.83	97.84	1.13	0.4
Sawdust	516.58	163.45	242.23	78.78	68.36	1.54	0.56
IFM /geo-foam	516.58	447.12	465.05	17.93	13.45	0.35	2.21
IFM /sawdust	516.58	461.65	467.15	5.5	10.63	0.11	2.97
CP	516.58	132.83	–	–	132.83	–	8.65
DMC	516.58	158.87	–	–	158.87	–	10.32
SC	516.58	196.72	–	–	196.72	–	12.44
SP	516.58	472.69	–	–	472.69	–	16.72

## Discussion

The developed numerical models were validated using Plaxis 8 for embankment supported by CP by the results of the full-scale tests and other numerical analyses, calibrated with experimental data, and conducted a sensitivity analysis to assess the impact of changes in key parameters on model outcomes. Furthermore, the other alternatives examined in this study were supported by existing literature and peer-reviewed studies, ensuring the validity and reliability of the models for evaluating the effectiveness of different embankment construction techniques on weak soils.

**Fig. 12:**
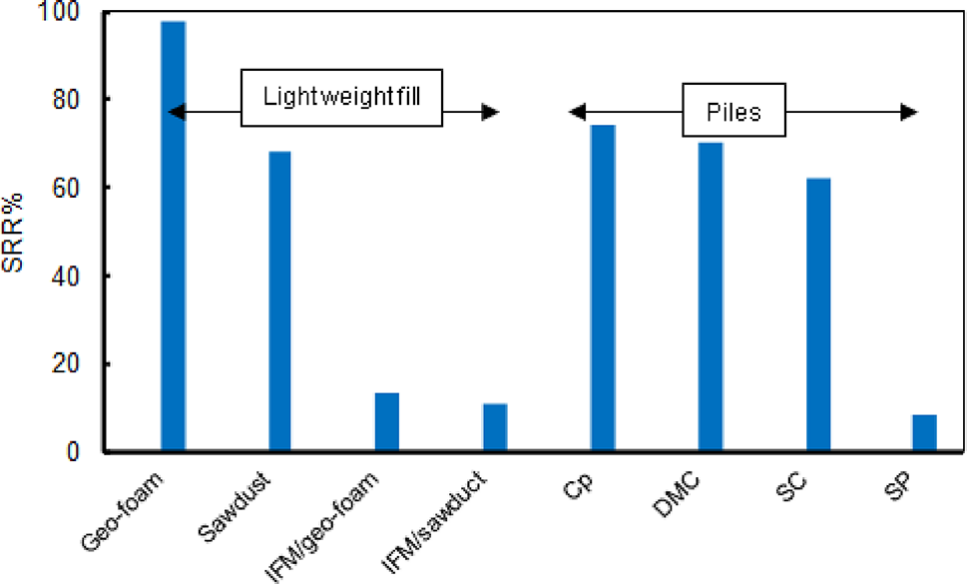
Comparison between the improvement factors obtained for different improvement methods.

The study revealed that although the outcomes of the different varied significantly, some of them showed convergence in their outcomes. This finding is promising for addressing issues commonly faced in incomplete projects or the use of unsuitable measures that contribute to soil instability. Figure 12 illustrates a comparative analysis of the settlement reduction factor for the multi-layer soil studied, reflecting all improvement efforts. Based on these findings, the optimal recommendation is to use geo-foam for constructing the embankment to its full-designated height, provided this approach does not result in unacceptable compressibility. Additionally, by analyzing the obtained results, it can be recommended that DMC is a preferred alternative to CP for constructing the embankments over soft soils, offering significant advantages over more than SP or SC. Employing cement-stabilized soil for making the DMC enhances its ability to handle the load-bearing capacity, the stress acting on the soil, the axial tensile force within the geosynthetic reinforcement layer, and settlement. This makes the effectiveness of the DMC close to CP while SC and SP improve drainage and compaction but have lower load capacity.

## Conclusions

Utility the 2D finite element software Plaxis 8, The behaviour of multilayer clayey soil under an embankment of lightweight filling materials such as mixed sawdust or geo-foam and that carrying an embankment of traditional fill material improved by deep replacement techniques like concrete piles (CP), deep-mixing columns (DMC), stone columns (SC), and sand piles (SP) were compared considering factors like stress distribution, pore water pressure, and settlement of the soil. The study’s conclusions are summarized as follows:

1. The use of lightweight materials led to a significant reduction in soil settlement and stress by 5–89% however, it also increased the compressibility of the embankment.
2. Initial filling material (IFM) limits surface deformation but leads to more settlement and higher stress on the soil.
3. Increasing the stiffness of deep replacement materials resulted in a decrease in settlement and stress in the surrounding soil, ranging from 8.5 to 74% and 44–88%, respectively.
4. Stone columns (SC) and sand piles (SP) showed faster dissipation of pore water pressure due to their higher permeability, contributing to faster consolidation of the surrounding clay soil. However deep mixing columns (DMC) provided the best balance between stiffness and cohesion, making them highly effective in reducing both settlements and stresses on the embankment.
5. The modulus of elasticity of the deep replacement materials affects the load transfer mechanism and over time, there is a noticeable reduction in the stress applied to the soil and the geosynthetic layer, while the load carried by the deep replacement (piles) increases.
6. Geosynthetic layers help manage stress, redistribute loads effectively and contribute to evenly distributing loads across the surface.

Future research on the effectiveness of lightweight material for filling the embankments or using the deep replacement to support them over various soil types and environmental circumstances is advised. Which must based on sustainability guidelines and environmental effects into account. Additionally, full-scale tests are recommended to determine which techniques are best for a certain soil or project. This will support the creation of useful guidelines for applying deep replacement techniques and lightweight materials.

## Data Availability

The datasets used and/or analysed during the current study available from the corresponding author on reasonable request. All data generated or analysed during this study are included in this published article. The datasets generated and/or analysed during the current study are not publicly available due [REASON WHY DATA ARE NOT PUBLIC] but are available from the corresponding author on reasonable request.
